# Prevalence of sealants in relation to dental caries on the permanent molars of 12 and 15-year-old Greek adolescents. A national pathfinder survey

**DOI:** 10.1186/1471-2458-11-100

**Published:** 2011-02-14

**Authors:** Constantine J Oulis, Elias D Berdouses, Eleni Mamai-Homata, Argyro Polychronopoulou

**Affiliations:** 1 Associate Professor, Department of Paediatric Dentistry Dental School, University of Athens, Athens, Greece; 2 Paediatric Dentist, Clinical Instructor, Department of Paediatric Dentistry, Dental School, University of Athens, Athens, Greece; 3 Associate Professors, Department of Preventive and Community Dentistry, Dental School, University of Athens, Athens, Greece

## Abstract

**Background:**

The use of sealants as an effective measure for the prevention of pit and fissure caries in children has been well documented by several studies; either they are used on an individual or on a public health basis. In order to plan and establish a national preventive program with sealants in a community, it is mandatory to know the epidemiological pattern of caries along with other variables influencing their use and effectiveness. Aims: To assess the utilization and distribution pattern of pit and fissure sealants on the first and second permanent molars of Greek adolescents and to evaluate whether the existing usage of sealants and some socio-demographic factors are correlated to caries prevalence on the population examined

**Methods:**

A stratified cluster sample of 2481 Greek adolescents was selected according to WHO guidelines (1224 twelve and 1,257 fifteen-year-old), living in urban and rural areas in 11 districts within the country. Five calibrated examiners carried out clinical examinations, recording caries experience at the dentine threshold (BASCD criteria) and presence or absence of sealants along with Socio-demographic indicators associated with oral health. Mann Whitney and Pearson's chi-square non parametric tests were utilized for assessing the data. The level of significance was p < 0.05.

**Results:**

Sealants utilization varied considerably within the different districts, with 8,3% of the 12 and 8,0% of the 15-year-old adolescents having at least one sealed molar. Sealants reduced DMFS scores by 11% in the 12-year-olds and by 24% in the 15-year-olds, while 15-year-old adolescents from rural areas had a statistically significant (p = 0.002) less chance of having sealants (71%) compared to children from urban areas. Girls had higher chance to receive sealants in both age groups (26% for the 12 and 19% for the 15-year-old) as well as patients that visited the dentist for prevention compared to those visiting the dentist because they thought they needed a restoration or because they were in pain.

**Conclusions:**

The finding that sealants reduced DMFS scores despite their very low utilization, along with the high prevalence of dental caries found on the occlusal surfaces of the posterior teeth of Greek adolescents, is calling for a national preventive program with sealants which could eliminate caries to a larger extent.

## Background

Although the overall caries rates have decreased considerably in most industrialized countries, the percentage of caries in pit and fissures compared to smooth surfaces has increased[1] making pits and fissures to constitute in some countries the most vulnerable sites raising the total DMFT. According to National center for health statistics in USA [2] the prevalence of dental caries increases with age, from 21% (6-11 year old) to 67% in adolescents (16-19 year old) with 90% of carious lesions found in occlusal surfaces of molars in children and young adults. In line with the above are the findings of a study by Whelton et al. [3], in which 81% of the DMFS of 4.0 and 72% of the DMFS of 6.1, in fluoridated and non-fluoridated areas respectively, occurs in pit and fissure surfaces of 15-year-old adolescents. Dental sealants are applied as a preventive measure to cover pit and fissures on the occlusal surfaces of children or teeth at risk for developing caries. The effectiveness of fissure sealants (FS) in preventing caries on pit and fissures of children has been well documented [4,5] and their effectiveness is dependent on the caries risk of the individuals [6] and the caries prevalence of the country [7]. In particular, studies have shown that the caries-free status of children 6-17 years of age has been associated with subsequent sealant placement [8].

Based on these findings and in an attempt to reduce caries on pit and fissures, several countries introduced the use of FS in school-based or public preventive programs with excellent results. According to Wendt et al. [9] and Parnell et al. [10], a structured fissure sealing programme is of great benefit to oral health of children since those who had no sealants had significantly poorer dental health than children who had all four first permanent molars sealed. In addition, it has been also suggested [11], that the overall less caries found in the Irish population might have been contributed to the existence of a pit and fissure sealant program delivered by the dental public health service in Ireland.

Some researchers believe that, FS can be effective in countries with DMFT below 2 and especially if the target is to lower the DMFT from 1.5 to 1.0 [12]. However, some other studies have shown that the higher the DMFT scores, the higher the caries reduction and caries free children when FS are used [13,14].

Based on the findings of the last National Oral Health survey in Greece[6] that most of the caries experience of the 12-year-old with a DMFS = 3.58 and of the 15-year-old with a DMFS = 5.36, was found on the occlusal surfaces of molar teeth a more tooth surface targeting preventive program with sealants could eliminate caries to a large extent. However, for such a sealant preventive program, some knowledge regarding the existing usage of sealants in relation to caries and the confounding factors influencing their use need to be answered, in order to determine the feasibility and appropriateness of this type of intervention at a National level.

Therefore the aim of this study was to assess the utilization and distribution pattern of pit and fissure sealants on the first and second permanent molars of Greek adolescents and to evaluate whether the existing usage of sealants and some socio-demographic factors are correlated to caries prevalence on the population examined.

## Methods

As part of a National pathfinder survey a stratified cluster sample of 2481 (1224 twelve and 1257 fifteen-year-old) adolescents of Greek nationality leaving in urban and rural areas, was selected according to the WHO guidelines [15].

The study covered two large cities, six counties, two islands in the Aegean Sea and one island in the Ionian Sea. Three communities with different socio-economic backgrounds were selected randomly within each of the large cities, while one urban and one rural community were selected randomly within each county or island. Therefore, the survey was conducted in 14 urban and 8 rural cites. Stratified random sampling was employed to select two schools from each city.

Five well-calibrated examiners assisted by one assistant as a recorder carried out the clinical examinations, which took place in the classrooms of the selected schools under standardized conditions recommended by the WHO. The examinations were carried out under artificial light using dental mirrors and the WHO CPITN periodontal probe. Cotton rolls and gauze were available for moisture control and removal of plaque when necessary. The recorded variables were caries experience, sealant's presence (regardless if it was total or partial), periodontal status, and oral hygiene level. Dental caries was recorded at the dentine threshold according to the agreed BASCD criteria and standards [16] set out in the BASCD trainers' pack [17]. Sociodemographic indicators associated with oral health such as: location (urban-rural), gender, parent's education level, type and mode of preventive measures and reason for visiting a dentist were collected via a questionnaire filled by the adolescent. A level of at least 85% for inter- and intra-examiner agreement was obtained for the recording of dental caries and sealant's presence in order for the calibration to end as successful.

### Statistical analysis

DMFS variable did not present Gaussian distribution and it received positive values only, the values were skewed positively and presented over dispersion. Therefore, the effect of this variable was analysed with generalized linear models that compensate for over dispersion namely the negative binomial regression analysis. The estimated coefficient was the incidence rate ratio (IRR). In order to evaluate the possibility of the presence of sealant even in one tooth logistic regression models were used. The estimated coefficient was odds ratio (OR) along with the 95% confidence interval and the p values. The comparisons were estimated against one of the categories that was considered baseline. Also, frequencies, proportions, χ^2 ^test and the non parametric tests Mann-Whitney and Kruskal-Wallis were used to describe and evaluate the distribution of sealants in the sample. The level of significance was set at p < 0.05. Patients that had never visited a dentist were excluded from calculation of the probability to receive sealants. The clinical examination and the registration of the dental condition of the individuals were possible after parental consent, permission from the Ministry of Education and the ethical approval of the committee of Research and Deontology in Dentistry of the University of Athens.

## Results

Table 1 presents the distribution of the sample according to district, DMFT and DMFS values, gender, location and reason for visiting a dentist as well as the contribution of molars to the DMFT. A very small percentage, less than 2%, of the sample in both age groups did not have any dental visit. The variation of the DMFT value was from 1.50 in the district of Attica to 2.87 in the district of Ioannina for the 12-year-old group and 2.35 in the district of Attica to 4.32 in the district of Achaia. The DMFT and DMFS values for the 12-year-old group were 2.05 and 3.58 respectively and for the 15-year-old were 3.19 and 5.36. The most common reason to visit the dentist was prevention followed by restoration while pain was the least common cause to visit but still accounting for 18.6% and 16.5% for the 12 and 15-year-old group respectively. About 20% of the sample came from rural areas and 80% from urban for both age groups. Caries free children accounted for 37.1% and 28.9% for the two age groups, respectively. First and second molar DMFT value was 1.62 for the 12 and 2.48 for the 15-year-old adolescents.

**Table 1:** Distribution of 12 and 15-year-old adolescents, according to district, DMFT and DMFS values, gender, location and reason for visiting a dentist

	12-year-old			15-year-old		
**District**	**N**	**DMFT (SD)**	**DMFS (SD)**	**N**	**DMFT (SD)**	**DMFS (SD)**

Attica*	160	1.50 (2.43)	2.69 (5.69)	150	2.35 (3.09)	3.69 (5.76)
Achaia	100	2.67 (3.19)	4.62 (7.01)	100	4.32 (4.13)	7.12 (8.50)
Evros	100	2.24 (2.64)	3.61 (4.80)	114	3.35 (3.37)	5.25 (6.08)
Thessaloniki	154	1.28 (1.78)	2.33 (4.30)	155	2.66 (3.20)	4.45 (6.08)
Ioannina	101	2.87 (2.97)	5.08 (8.76)	105	3.76 (3.05)	6.11 (6.10)
Kastoria	101	2.34 (2.25)	4.40 (5.15)	104	3.26 (3.72)	6.34 (8.72)
Keffalonia	101	1.58 (2.16)	2.60 (4.76)	101	2.68 (2.92)	4.31 (5.40)
Larisa	101	2.38 (2.70)	3.67 (4.82)	105	3.54 (3.75)	5.53 (6.74)
Lesvos	100	2.20 (2.04)	3.73 (4.34)	108	3.08 (2.77)	4.87 (4.79)
Naxos	102	2.26 (2.75)	3.89 (6.30)	104	3.75 (4.16)	6.35 (7.96)
Chania	104	1.97 (2.05)	3.97 (5.07)	111	2.93 (3.43)	6.13 (9.20)

**Total**	**1224**	**2.05 (2.50)**	**3.58 (5.64)**	**1257**	**3.19 (3.45)**	**5.36 (6.96)**

**Gender**	**N (%)**			**N (%)**		

**Male**	580 (47.4)			549 (43.7)		
**Female**	644 (52.6)			708 (56.3)		
**No Dental Visit**	19 (1.6)			18 (1.4)		

**Reason for Dental Visit**						
Pain	224 (18.6)			205 (16.5)		
Restoration	424 (35.2)			475 (38.3)		
Prevention	557 (46.2)			559 (45.1)		

**Rural**	248 (20.3)			252 (20.0)		
**Urban**	976 (79.7)			1005 (80.0)		

**Children with caries**	770 (62.9)			894 (71.1)		
**DMFT of molars**	1.62			2.48		

In table 2, the distribution of sealant and caries prevalence by age, tooth type and surface of first and second permanent molars of Greek adolescents is presented. Four percent of the maxillary and five percent of the mandibular 1^st ^permanent molars of the 12-year-old group had sealants while 4% of the 1^st ^permanent molars in both arches, of the 15-year-old group were sealed. Eight percent of the total sample in both age groups, presented with at least one posterior tooth sealed. First molars were sealed more often than second molars in both age groups. Sealants were found in 8.3% and 1.4% of the first and second molars in 12 and 7.4% and 2.2% in 15-year-old respectively.

**Table 2:** Distribution of sealant prevalence of the first and second permanent molars of Greek 12 and 15-year-old adolescents

	12-year-old							
	**16**	**26**	**36**	**46**	**17**	**27**	**37**	**47**
	**N (%)**	**N (%)**	**N (%)**	**N (%)**	**N (%)**	**N (%)**	**N (%)**	**N (%)**

**Occlusal**	51 (4.2)	54 (4.4)	65 (5.3)	61 (5.0)	4 (0.5)	3 (0.3)	10 (1.0)	8 (0.8)
**Lingual**	47 (3.8)	54 (4.4)			4 (0.5)	3 (0.3)		
**Buccal**			61 (5.0)	58 (4.7)			10 (1.0)	8 (0.8)
**Teeth with caries**	401 (32.8)	390 (31.9)	468 (38.2)	476 (38.9)	33 (4.0)	26 (3.0)	94 (9.4)	90 (7.4)
**Teeth without caries**	823 (67.2)	834 (68.1)	756 (61.8)	748 (61.1)	791 (96.0)	835 (97.0)	904 (90.6)	912 (74.5)
**Teeth with Sealants**	52 (4.2)	54 (4.4)	65 (5.3)	61 (5.0)	4 (0.5)	3 (0.3)	10 (1.0)	8 (0.7)

**Patients with at least one sealant: **102 (8.3)								

	**15-year-old**							

**Occlusal**	55 (4.4)	52 (4.1)	50 (4.0)	55 (4.4)	13 (1.0)	10 (0.8)	14 (1.1)	15 (1.2)
**Lingual**	52 (4.1)	49 (3.9)			13 (1.0)	11 (0.9)		
**Buccal**			48 (3.8)	54 (4.3)			13 (1.0)	15 (1.2)
**Teeth with caries**	512 (40.7)	488 (38.8)	586 (46.6)	607 (48.3)	158 (12.6)	194 (15.4)	300 (23.9)	274 (21.8)
**Teeth without caries**	745 (59.3)	769 (61.2)	671 (53.4)	650 (51.7)	1099 (87.4)	1063 (84.6)	957 (76.1)	983 (78.2)
**Teeth with sealants**	55 (4.4)	52 (4.1)	50 (4.0)	55 (4.4)	13 (1.0)	11 (0.9)	14 (1.1)	15 (1.2)

**Patients with at least one sealant: **100 (8.0)								

Sealants utilization varied considerably between the different districts (table 3), with the two most populated districts of the country, Attica and Thessaloniki showing higher prevalence of sealants use compared to other districts. Multifactorial logistic regression analysis was used to evaluate the effect of different factors on the probability of a child to receive sealant. Children in all districts presented a lower probability (< 1) of receiving sealants compared to Attica, except of the district of Larisa in the 12-year-old group (1.17) and district of Lesvos (1.45) in the 15-year-old group. Fifteen year-old adolescents from rural areas had a statistically significant (p = 0.002) less chance (71%) of having sealants compared to adolescents from urban areas. Girls had higher chance to receive sealants in both age groups (26% for the 12 and 12% for the 15-year-old), but not with statistically significant difference.

**Table 3:** Variables predicting sealant placement on permanent molars of 12 and 15-year-old Greek adolescents (Multifactorial - Logistic Regression Analysis)

	12-year-old			15-year-old		
**District**	**Odds Ratio**	**95% C.I**.	**P-value**	**Odds Ratio**	**95% C.I**.	**P-value**

Attica*	1			1		
Achaia	0.37	(0.12, 1.14)	0.084	0.33	(0.11, 1.02)	0.053
Evros	0.31	(0.11, 0.84)	**0.022^1^**	0.44	(0.19, 1.02)	0.055
Thessaloniki	0.63	(0.33, 1.19)	0.155	0.41	(0.19, 0.88)	**0.021^1^**
Ioannina	0.41	(0.16, 1.07)	0.068	0.44	(0.19, 1.05)	0.066
Kastoria	0.52	(0.23, 1.18)	0.119	0.20	(0.07, 0.59)	**0.004^1^**
Keffalonia	0.26	(0.10, 0.72)	**0.009^1^**	0.60	(0.24, 1.53)	0.288
Larisa	1.17	(0.58, 2.35)	0.655	0.45	(0.19, 1.07)	0.071
Lesvos	0.38	(0.14, 1.05)	0.063	1.45	(0.68, 3.10)	0.335
Naxos	0.26	(0.09, 0.79)	**0.018^1^**	0.53	(0.20, 1.38)	0.194
Chania	0.16	(0.04, 0.07)	**0.015^1^**	0.44	(0.17, 1.16)	0.097

**Mother's Educational Levels**						
College*	1			1		
High	0.60	(0.39, 0.94)	**0.026^1^**	0.61	(0.41, 1.01)	0.055
Elementary	0.23	(0.09, 0.58)	**0.002^1^**	0.54	(0.26, 0.95)	**0.035^1^**

**Reason Visiting a Dentist**						
Prevention*	1			1		
Restoration	1.37	(0.86, 2.18)	0.184	0.76	(0.47, 1.23)	0.264
Pain	0.43	(0.21, 0.92)	**0.029^1^**	0.57	(0.29, 1.10)	0.092

**Urban/Rural**						
Rural				0.29	(0.13-0.63)	**0.002^1^**
Urban*				1		

**Gender**						
Male	1			1		
Female	1.26	(0.84, 1.89)	0.272	1.12	(0.74, 1.68)	0.603

* Baseline Category

^1^Variable with statistical difference compared with the baseline

In the 15-year-old group, patients that visited the dentist because they thought they needed a restoration or they were in pain had a reduced chance of receiving a sealant by 24% and 43% respectively, compared to patients that visited the dentist for prevention but not in a statistically significant level. In the 12-year-old group, patients that visited the dentist because they thought they needed a restoration had a not statistically significant increased chance (37%) of receiving a sealant compared to patients that visited for prevention. On the contrary, patients that visited because of pain had a statistically significant reduced chance (57%) of receiving sealant (p = 0.029) compared to patients that visited the dentist for prevention. Also, as the educational level of parents increases the probability of their children to receive sealant increases statistically significant.

Table 4 presents the distribution of caries in the different surfaces of the teeth. It was found that 16.8% were in the anterior teeth and 83.2% in the posterior teeth of the 12-year-old group while the respective values for the 15-year-old group were 13.2% for the anterior and 86.9% for the posterior teeth. Caries located in pit and fissures (occlusal surface, lingual of maxillary and buccal of mandibular molars) accounted for 56.2% of the total caries on 12-year-old and 58.0% of the 15-year-old children or for 67.6% and 66.7% of the caries of the posterior surfaces in the two age groups respectively.

**Table 4:** Distribution of caries in 12 and 15-year-old adolescents according to surface of anterior and posterior teeth

	12-year-old		
	**Anterior** **N (%)**	**Posterior** **N (%)**	**Total**

Occlusal		1900 (43.3)	1900 (43.3)
Mesial	249 (5.7)	535 (12.2)	784 (17.9)
Distal	157 (3.6)	323 (7.4)	480 (10.9)
Lingal	206 (4.7)	413 (9.4)	619 (14.1)
Buccal	124 (2.8)	483 (11.0)	607 (13.8)
**Total**	**736 (16.8)**	**3654 (83.2)**	**4390 (100)**

**DMFS**	**0.60**	**2.98**	**3.58**

Pit & Fissures out of total caries			2469 **(56.2)**
Pit & Fissures out of caries in posterior teeth			2469 **(67.6)**

	**15-year-old**		

Occlusal		3131 (46.4)	3131 (46.4)
Mesial	321 (4.8)	883 (13.1)	1204 (17.8)
Distal	172 (2.6)	638 (9.5)	810 (12.0)
Lingal	211 (3.2)	591 (8.8)	802 (12.0)
Buccal	177 (2.6)	619 (9.2)	796 (11.8)
**Total**	**881 (13.1)**	**5862 (86.9)**	**6743 (100)**

**DMFS**	**0.70**	**4.66**	**5.36**

Pit & Fissures out of total caries			3911 **(58.0)**
Pit & Fissures out of caries in posterior teeth			3911 **(66.7)**

The effect of sealant on DMFS was evaluated with negative binomial regression analysis and it was found that sealants reduced caries prevalence by 11% in the 12-year-old group (not statistically significant) and by a statistically significant level of 24% in the 15-year-old group (table 5).

**Table 5:** Effect of sealant presence on DMFS of 12 and 15-year-old Greek adolescents (Negative Binomial Regression)

	12-year-old			15-year-old		
**Sealants**	**IRR**	**95% C.I**.	**P-value**	**IRR**	**95% C.I**.	**P-value**

No*	1			1		
Yes	0.89 (11%)	(0.65, 1.22)	0.467	0.76 (24%)	(0.57, 1.00)	**0.049^1^**

* Baseline Category

^1 ^Variable with statistical difference compared with the baseline

## Discussion

This study, as part of the first oral health National pathfinder survey of the Hellenic population, was aimed to assess the utilization and the distribution pattern of sealant use in 12 and 15-year-old adolescents in relation to their caries prevalence and the influence of several sociodemographic parameters.

Based on the findings of this study, sealant utilization in adolescents throughout the country was very low (8%) and most likely this might be attributed to lack of awareness of the public and that the dentists have not been convinced on the usefulness and effectiveness of sealants on caries prevention. This hypothesis is supported from the findings of another study conducted in Greece aiming to investigate the dentist's beliefs on sealant use [18]. According to this study, although 68.8% of the general dental practitioners in Greece believe in prevention and 59.0% believe in the effectiveness of sealants only 30.3% apply them in practice due to a number of concerns they raise about their use. The expressed concerns such as "parents do not pay" or "unaware how to use them" or "other preventive measures like oral hygiene are sufficient to prevent caries" show that there is a serious lack of the appropriate knowledge between general dental practitioners in Greece, regarding sealant effectiveness [19]. Dentist's reservations of sealant effectiveness and unawareness on how to use them might have been the reasons for not being successful in persuading the parents to accept sealants. On the other hand, dentists are very important in educating parents on carries prevention issues and they can increase sealant use in the population. This occurs because parents are more inclined to accept advice from them on caries prevention since dentists are the most appropriate source of information on such issues [19].

The low prevalence of sealants on both ages might also explains in part the high prevalence of caries found (almost 2-fold) in Greek adolescents compared to other European countries. Especially when, most of the caries in the two age groups (83 and 87%) was found on the posterior teeth and the majority of it (67.6 and 66.7%) was located on pit and fissures that can be mostly benefited and caries might be prevented by the use of sealants.

Despite the low sealant use, sealant placement was associated with caries reduction in both age groups. This reduction was not statistically significant (11%) in the 12-year-old group but increased to a statistically significant 24% for the 15-year-old group. Our findings, on the association of sealant's prevalence and DMFS reduction are in agreement with other similar studies conducted in other European countries, in which the sealant implementation resulted in significant lower DMFS values [20] and the degree of caries reduction (16-60%) was dependent on the prevalence of sealants and the caries level of the population [20,21]. In Germany [21], after the introduction of sealants in a range of 16-45% of the population, DMFT index decreased from 3.54 in 1997 to 1.24 in 2004.

However, there are other countries like Denmark, where although sealant's prevalence was very high, 2/3 of 15-year-old Danish children had at least one sealed molar, there was no statistical significant association of caries reduction and sealant presence [22]. According to our opinion, this might be attributed to the fact that sealants were applied on children with a very low DMFS value (2.97 compared to 5.36 of ours) and although there was such a high prevalence of sealants, it could not be further reduced This explanation might also be valid for not finding any statistically significant difference in caries reduction in the 12-year-old group of our study. This group presented with a rather low DMFS value of 3.58 which in conjunction with the low prevalence of sealants probably were not adequate to produce any statistically significant difference.

According to some researchers, sealants can be effective in countries with DMFT below 2 [12], while some others have shown that the higher the DMFT scores, the higher the caries reduction and the number of caries free children [13,23]. It seems that between the two factors, the DMFT value has a stronger contributing effect on caries reduction than the prevalence of sealants in the population.

Our data also showed a very large inter-district variation in sealants use and a significantly increased possibility of children of larger cities and urban areas to receive sealants compared to children from smaller cities or rural areas. This finding can be attributed to a different attitude of the population on sealant efficacy, a better access to professionally provided preventive oral health services [22,24] and/or that rural areas generally have fewer dentists per population and more poverty resulting in lower access and utilization of dental care [25]. It is also known that there are striking disparities in dental disease prevalence among people based on socio-demographic characteristics, such as income, location, and parental educational level [24]. Moreover, studies have shown that the percentage of children with dental sealants is directly related to the community's SES [26] and that children from low income and minority families have fewer dental visits, and fewer protective sealants [27].

The solution to the problem for a population with such a low sealant prevalence, high DMFS values and large inter-district variation in sealant use seems to be a school-based or a national sealant programme, aiming to increase sealant's prevalence up to 40-50%. Considering the findings from other similar programs in other countries, in which a caries reduction of up to 60% was succeeded after 5 years of sealant application [28], a target of reducing our adolescent's DMFS value by 50% after 5 years, seems to be very logical.

Therefore, a school-based or national sealant preventive programme is needed to be organized and implemented in Greece, with special emphasis on increasing the knowledge and awareness of dentists and the public on sealant usefulness in order to benefit all children but mainly the most disadvantageous group of children that need it the most.

## Conclusion

• The survey indicated that there was a low utilization of sealants in Greece compared to other European countries and their distribution varied considerably between the different districts, showing a significant higher use in urban than in rural areas, in females than in males and in children from parents with higher educational level.

• Sealants, even in low prevalence, contributed to a significant reduction of caries in a high DMFT population suggesting that they are more effective in high DMFT populations. Also, DMFT of the population is more important for the sealant efficiency than sealant prevalence.

• Patients that visited dentists because of pain or lived in rural areas had reduced chances to receive sealants

• The high prevalence of caries found in the posterior teeth, along with the low sealant use in Greek adolescents, necessitate the establishment of a more targeted preventive program with better and more effective oral health education, which will increase the sealant use and thus can greatly reduce caries on their permanent posterior teeth.

## Competing interests

The authors declare that they have no competing interests.

## Authors' contributions

CJO: conceived the study, co-organised the epidemiological survey, made the analysis and interpretation of data, discussed the quality of the paper, approved and corrected the final draft. EDB: drafted the manuscript, organised the data selection. EMH: participated in drafting the paper and discussing the relevant literature in caries epidemiology. AP: contributed in the design and sampling of the study and checked the statistical analysis.

## Pre-publication history

The pre-publication history for this paper can be accessed here:

http://www.biomedcentral.com/1471-2458/11/100/prepub
